# Refractory Gastric Antral Vascular Ectasia as a Sentinel for "Burned-Out" Metabolic Dysfunction-Associated Steatotic Liver Disease and Rapid Hepatocellular Carcinoma Progression in a Post-transcatheter Aortic Valve Implantation Dialysis Patient

**DOI:** 10.7759/cureus.105316

**Published:** 2026-03-16

**Authors:** Willy Stéphane Kengne, Baro Deressa, Luc Lasser

**Affiliations:** 1 Department of Gastroenterology and Hepatology, Centre Hospitalier Universitaire (CHU) Brugmann, Brussels, BEL

**Keywords:** cryptogenic cirrhosis, diagnostic errors, liver steatosis, multimorbidity, vascular ectasia

## Abstract

Gastric antral vascular ectasia (GAVE) is challenging to manage in multimorbid patients, where etiology varies between cirrhosis, autoimmune disease, or chronic kidney disease (CKD). We report a 76-year-old man with transcatheter aortic valve implantation (TAVI), hemodialysis, and past alcohol abuse presenting with refractory GAVE. Baseline imaging in 2021 showed metabolic dysfunction-associated steatotic liver disease (MetALD) with advanced fibrosis and severe steatosis (S3). Two years later (April 2023), during acute bleeding, despite six sessions of endoscopic ablation, hemostasis was elusive. At this stage, elastography showed deceptive radiological evidence of complete steatosis regression (S0) with stable fibrosis. This "pseudo-normalization", likely driven by fibrosis replacing fat and tumor-induced lipid mobilization, masked the underlying pathology. Eight months post-bleeding (December 2023), clinical deterioration revealed decompensated cirrhosis and a massive 6.7 cm hepatocellular carcinoma (HCC). Diagnostic obscurity caused by steatosis resolution in "burned-out" MetALD can be fatal. In this patient, the refractory nature of the GAVE, rather than its mere presence, served as a crucial sentinel sign for occult malignancy and advancing portal hypertension. The patient was referred for palliative care and remains under supportive management.

## Introduction

Gastric antral vascular ectasia (GAVE), often referred to as "watermelon stomach", accounts for approximately 4% of non-variceal upper gastrointestinal bleeding [1]. Its pathophysiology is dual, associated with either portal hypertension (30% of cases) [2] or systemic conditions such as autoimmune disease and chronic kidney disease (CKD) [3]. In the elderly population, distinguishing GAVE from Heyde's syndrome (angiodysplasia associated with aortic stenosis) is critical for appropriate therapeutic targeting.

Managing GAVE in multimorbid patients presents a significant clinical challenge. This article presents a case of refractory GAVE in a patient with a complex clinical convergence of conditions consisting of post-transcatheter aortic valve implantation (TAVI), dialysis, and hepatopathy. Distinguishing the primary driver of GAVE among Heyde's syndrome, uremic vasculopathy, and portal hypertension is clinically vital, as it dictates the therapeutic strategy: valve replacement, intensive dialysis, or portal decompression (transjugular intrahepatic portosystemic shunt (TIPS)), respectively.

In this patient, resistance to endoscopic therapy served as a clinical "red flag" for a specific pathophysiological entity known as "malignant portal hypertension". Malignant portal hypertension occurs when tumor burden causes direct vascular compression, arterioportal shunting, or microvascular invasion, leading to a rapid and localized rise in portal pressure [4]. Furthermore, this case highlights the diagnostic trap of "burned-out non-alcoholic steatohepatitis (NASH)". This phenomenon occurs when advancing fibrosis paradoxically leads to the disappearance of hepatic steatosis because fibrotic tissue replaces fat-storing hepatocytes, masking the underlying disease severity on standard imaging.

## Case presentation

A 76-year-old man was admitted for severe symptomatic anemia in April 2023. His medical profile was characterized by significant multimorbidity. His cardiovascular history included severe aortic stenosis treated with TAVI in 2022; he was on daily aspirin 75 mg, which was suspended upon admission. Regarding renal status, the patient had end-stage renal disease (ESRD) and had been on hemodialysis since February 2022. His metabolic and toxic history included type 2 diabetes mellitus diagnosed in 2007 and a history of severe chronic alcohol consumption (approximately 50 units/week), which ceased two years prior to admission.

Baseline laboratory values at admission and subsequent deterioration are summarized in Table 1.

**Table 1:** Laboratory result summary INR: international normalized ratio; AST: aspartate aminotransferase; ALT: alanine aminotransferase; AFP: alpha-fetoprotein

Category	Parameter	Baseline (April 2023)	Clinical deterioration (December 2023)	Reference range
Hematology	Hemoglobin	5.8 g/dL	8.2 g/dL	13.5-17.5 g/dL
	Platelets	145 g/L	<100 g/L	150-450 g/L
	INR	1.1	1.6	0.8-1.2
Biochemistry	Bilirubin (total)	14 µmol/L	36 µmol/L	2-21 µmol/L
	Albumin	32 g/dL	24 g/dL	37-49 g/L
	Alkaline phosphatase	72 UI/L	185 UI/L	45-105 UI/L
	AST	45 UI/L	88 UI/L	1-31 UI/L
	ALT	32 UI/L	52 UI/L	5-35 UI/L
	AFP	4.2 ng/mL	450 ng/mL	<10 ng/mL

Baseline laboratory values at admission revealed the following: hemoglobin 5.8 g/dL, platelets 145 G/L, international normalized ratio (INR) 1.1, bilirubin 0.8 mg/dL, and albumin 3.2 g/dL. Liver function tests remained relatively quiescent, with alkaline phosphatase ranging from 57 to 96 UI/L and transaminases between 19 and 60 UI/L.

In March 2021, while the patient was still consuming alcohol, transient elastography (FibroScan) documented a metabolic dysfunction-associated steatotic liver disease (MetALD) profile with advanced fibrosis (liver stiffness measurement (LSM): 14.5 kPa) and severe steatosis (controlled attenuation parameter (CAP): 310 dB/m, S3).

**Figure 1:**
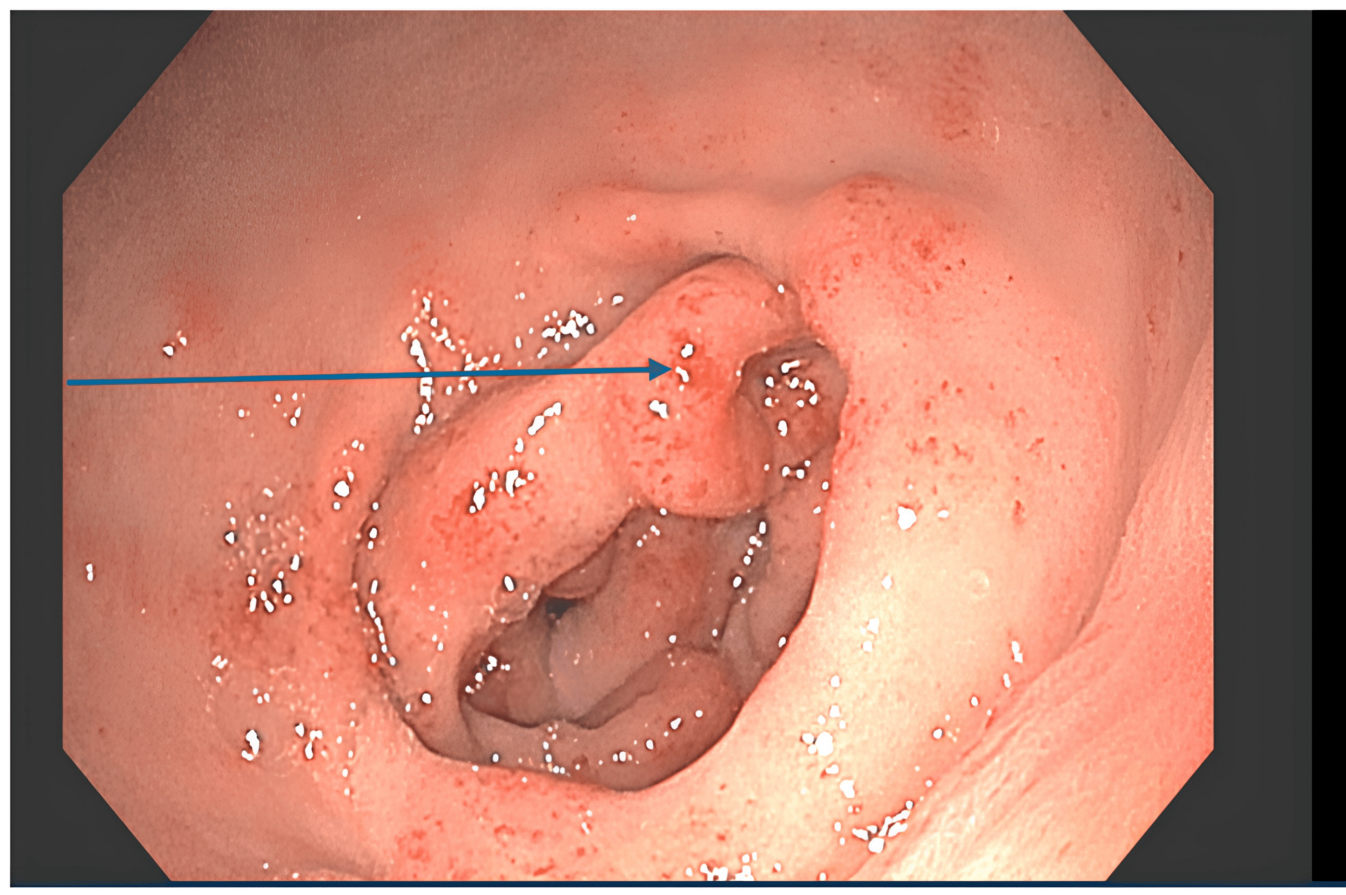
Endoscopic view of the gastric antrum showing diffuse vascular ectasia with linear red streaks (watermelon stomach). The blue arrow indicates an area of active oozing resistant to thermal ablation

Gastroscopy in April 2023 confirmed active, oozing GAVE (Figure 1). Due to high surgical risk and contraindications for TIPS given the cardiac history, endoscopic therapy was chosen. Six sessions of thermal ablation using a bipolar probe (BiCap) were performed between April and June 2023. The bipolar probe was chosen over argon plasma coagulation (APC) to allow for deeper coaptation of the larger ectatic vessels observed, as well as to minimize the risk of gastric distension in this fragile patient. Despite this aggressive management, hemostasis was never achieved, and the patient remained dependent on recurrent red blood cell transfusions. Given the active bleeding and uremic coagulopathy, a liver biopsy was deemed too risky.

A diagnostic re-evaluation in June 2023 proved misleading. Abdominal ultrasound showed a homogeneous liver with no focal lesions. Transient elastography indicated F3 fibrosis but with radiological evidence of complete steatosis regression (CAP: <200 dB/m, S0). A computed tomography (CT) scan was withheld at this stage due to concerns regarding contrast-induced nephrotoxicity in a patient with residual diuresis. This apparent improvement in steatosis (S3 to S0) was misinterpreted as a positive outcome of alcohol cessation, leading clinicians to attribute the GAVE primarily to uremic vasculopathy.

**Figure 2:**
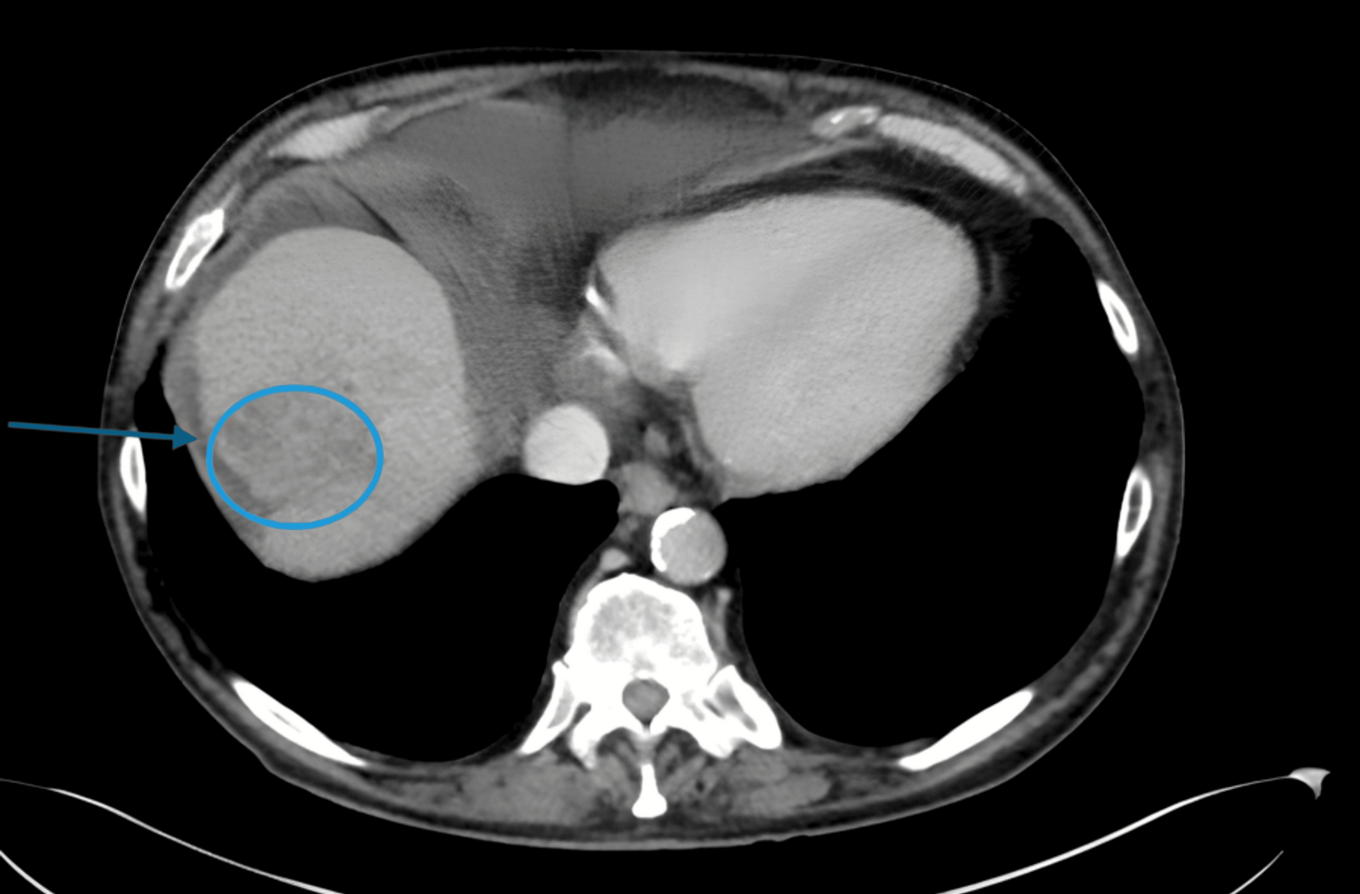
Contrast-enhanced abdominal CT scan (arterial phase) revealing a large 67 mm heterogeneous mass in liver segment VIII (indicated by blue circle and line), consistent with hepatocellular carcinoma in a dysmorphic liver CT: computed tomography

However, in December 2023, the patient's condition deteriorated with the onset of ascites and thrombocytopenia (<100 G/L). Liver function tests worsened, showing the following: bilirubin 2.1 mg/dL, albumin 2.4 g/dL, and INR 1.6. Alpha-fetoprotein (AFP) levels were elevated at 450 ng/mL. A contrast-enhanced CT scan was performed, revealing a dysmorphic liver with caudate lobe hypertrophy and a 67 mm heterogeneous mass in segment VIII consistent with hepatocellular carcinoma (HCC) (Figure 2).

While direct portal vein invasion was not visualized, the tumor mass effect in a dysmorphic liver suggested significant hemodynamic alteration. Concurrently, a control FibroScan showed massive progression to cirrhosis with an LSM of 40 kPa and a persistently low CAP of 190 dB/m. We acknowledge that the extreme rise in LSM (40 kPa) likely reflects a composite of advanced fibrosis, hepatic congestion from right heart failure, and the mass effect exerted by the large tumor. The patient was classified as Barcelona Clinic Liver Cancer (BCLC) stage D. He is currently alive and receiving best supportive care under the palliative team.

## Discussion

This case exemplifies the diagnostic dilemma inherent in the convergence of ESRD, aortic stenosis, and liver disease. While GAVE is prevalently associated with cirrhosis, it is also frequently reported in hemodialysis patients due to uremic platelet dysfunction and heparinization [3]. Differentiating the etiology is crucial as it dictates therapy: TIPS is preferred for portal hypertension-driven GAVE, whereas endoscopic ablation is standard for non-cirrhotic cases [5]. In our patient, the key discriminator was the refractoriness to therapy. Studies suggest that GAVE in cirrhotic patients has a significantly higher re-bleeding rate compared to non-cirrhotic causes [6]. We propose that absolute resistance to thermal ablation (defined as transfusion dependence despite >4 sessions) in a multimorbid patient should be considered a specific surrogate marker for severe portal hypertension, warranting invasive investigation regardless of renal risks.

The most significant learning point of this case is the misleading normalization of hepatic steatosis. The evolution from S3 (severe steatosis) to S0 (absence of steatosis) observed here is consistent with "burned-out NASH/MetALD". This term describes the regression of hepatic fat content that occurs as fibrosis progresses to bridging fibrosis or cirrhosis. Pathophysiologically, as the liver architecture becomes distorted and vascular resistance increases (capillarization of sinusoids), hepatocytes lose the metabolic capacity to accumulate triglycerides [7,8].

Distinct from this chronic fibrotic mechanism, acute systemic factors also accelerated lipid mobilization in our patient. The catabolic state induced by dialysis and occult malignancy accelerates lipid mobilization. Chronic hemodialysis induces a pro-inflammatory state characterized by elevated cytokines (IL-6, TNF-alpha), which promotes lipolysis and muscle wasting [9]. Furthermore, the tumor microenvironment in HCC exacerbates this by secreting lipid-mobilizing factors to fuel rapid neoplastic growth, effectively "stripping" the surrounding liver parenchyma of its fat stores [10].

The discovery of a 67 mm HCC only six months after a negative ultrasound highlights the limitations of sonography in this population. It is unlikely the tumor grew from undetectable to 67 mm in this short interval; rather, the regression of steatosis rendered the liver parenchyma isoechoic, reducing the sonographic contrast needed to detect the tumor [11]. The failure to detect dysmorphic features in June highlights the limitations of ultrasonography in obese or dialysis patients, where bowel gas and poor acoustic windows can obscure architectural distortion, making this modality highly observer-dependent. The rapid progression to HCC also highlights the synergistic toxicity of MetALD. Recent consensus data indicate that patients with MetALD have a higher risk of HCC compared to NAFLD alone [12]. The mechanism likely involves persistent insulin resistance driving insulin-like growth factor-1 (IGF-1) pathways, combined with epigenetic changes from prior alcohol exposure [13].

The rapid clinical progression suggests that the refractory GAVE was likely the clinical expression of a hemodynamic shift. While uremia and previous aortic stenosis (Heyde's physiology) undoubtedly contributed to the bleeding risk, the refractoriness to ablation suggests an acute elevation in portal pressure. The expanding tumor burden in a cirrhotic liver can rapidly alter hepatic outflow [14], potentially rendering endoscopic therapy futile long before the mass is radiologically visible on non-contrast imaging.

## Conclusions

In multimorbid patients with ESRD and chronic liver disease, the onset of refractory GAVE represents a strong clinical "red flag". The disappearance of hepatic steatosis (S3 to S0) in MetALD is a hallmark of "burned-out" disease and advanced fibrosis, not recovery. This case suggests that refractory GAVE can act as a sentinel for occult malignancy and advancing portal hypertension. Given the risks of contrast-induced nephropathy with CT and nephrogenic systemic fibrosis (NSF) with older gadolinium agents, we recommend prioritizing safer modalities such as non-contrast magnetic resonance imaging (MRI) (using diffusion-weighted sequences) for surveillance in this high-risk population, as standard ultrasound may be insufficient.
